# Residue substitution enhances the immunogenicity of neoepitopes from gastric cancers

**DOI:** 10.20892/j.issn.2095-3941.2021.0022

**Published:** 2021-11-24

**Authors:** Huahui Yu, Jieyu Li, Yuan Yuan, Yu Chen, Jingwen Hong, Chunmei Ye, Wansong Lin, Huijing Chen, Zengqing Guo, Bo Li, Yunbin Ye

**Affiliations:** 1 The School of Basic Medical Sciences, Fujian Medical University, Fuzhou 350122, China; 2 Laboratory of Immuno-Oncology, Fujian Cancer Hospital & Fujian Medical University Cancer Hospital, Fuzhou 350014, China; 3 Fujian Key Laboratory of Translational Cancer Medicine, Fuzhou 350014, China; 4 BGI-Shenzhen, Shenzhen 518083, China; 5 BGI Education Center, University of Chinese Academy of Sciences, Shenzhen 518083, China; 6 Department of Oncology, Fujian Cancer Hospital & Fujian Medical University Cancer Hospital, Fuzhou 350014, China

**Keywords:** Gastric cancer, bioinformatics, neoepitope, residue substitution, immunotherapy

## Abstract

**Objective:**

Neoantigens arising from gene mutations in tumors can induce specific immune responses, and neoantigen-based immunotherapies have been tested in clinical trials. Here, we characterized the efficacy of altered neoepitopes in improving immunogenicity against gastric cancer.

**Methods:**

Raw data of whole-exome sequencing derived from a patient with gastric cancer were analyzed using bioinformatics methods to identify neoepitopes. Neoepitopes were modified by P1Y (the first amino acid was replaced by tyrosine) and P2L (the second amino acid was replaced by leucine). T2 binding and stability assays were used to detect the affinities between the neoepitopes and the HLA molecules, as well as the stabilities of complexes. Dendritic cells (DCs) presented with neoepitopes stimulated naïve CD8^+^ T cells to induce specific cytotoxic T lymphocytes. ELISA and carboxyfluorescein succinimidyl ester were used to detect IFN-γ and TNF-α levels, and T cell proliferation. Perforin was detected by flow cytometry. The cytotoxicity of T cells was determined using the lactate dehydrogenase assay.

**Results:**

Bioinformatics analysis, T2 binding, and stability assays indicated that residue substitution increased the affinity between neoepitopes and HLA molecules, as well as the stabilities of complexes. DCs presented with altered neoepitopes stimulated CD8^+^T cells to release more IFN-γ and had a greater effect on promoting proliferation than wild-type neoepitopes. CD8^+^T cells stimulated with altered neoepitopes killed more wild-type neoepitope-pulsed T2 cells than those stimulated with wild-type neoepitopes, by secreting more IFN-γ, TNF-α, and perforin.

**Conclusions:**

Altered neoepitopes exhibited greater immunogenicity than wild-type neoepitopes. Residue substitution could be used as a new strategy for immunotherapy to target neoantigens.

## Introduction

In recent years, antibodies against PD-1, a key immune checkpoint inhibitor, have provided an important advance in tumor immunotherapy, and have been used in the treatment of a variety of tumors^1–5^. However, their clinical efficacy remains unsatisfactory because of various reasons, of which the neoantigen burden in the tumor is one of the key factors^6^.

Tumor antigens are classified into tumor-associated antigens and tumor-specific antigens, including oncovirus antigens and neoantigens generated by somatic mutations^7^. Unlike tumor-associated antigens, tumor neoantigens are not affected by immune tolerance^8,9^. The peripheral blood contains a large number of antigen-specific T cells against tumor neoantigens. Tumor-associated antigen-specific T cells with high affinities can be deleted during thymus development; therefore, the tumor-associated antigen-specific T cells in peripheral blood have low affinities for tumor-associated antigen epitopes^10,11^. However, neoantigen-specific T cells in the peripheral blood usually have high affinities for neoantigens^12^. Neoantigen specific T cells are more sensitive to neoantigen stimulation and suitable for immunotherapy when using tumor antigens.

There are 2 types of neoantigen-derived gene mutations: single nucleotide variants (SNVs) and insertions or deletions (InDels). SNVs, manifested as base replacements, are the most common mutations^13^. Because SNVs are easy to compare with the human reference genome, the detection of SNVs using next-generation sequencing is highly accurate; however, the reads carrying InDels in the sequencing results have a low coverage, which makes it difficult to detect InDels^14^. Two melanoma clinical trials in 2017 showed that neoantigen peptide vaccines and RNA vaccines derived from SNVs effectively stimulated immune responses in tumor patients^15,16^. Neoantigens derived from SNVs were therefore selected in this study. Whole-exon sequencing was performed on tumor tissues and peripheral blood from tumor patients^17–19^, and the somatic mutations^20^ were selected by comparing the sequencing results of tumor tissues and peripheral blood. MuPeXI^21^ was used to analyze neoantigens derived from SNVs from somatic mutations, and a comprehensive bioinformatics analysis was performed on neoantigens obtained from the analysis. Bioinformatics analyses included the affinity of the peptide to the antigen peptide transporter (TAP) and the affinity of the peptide to the T cell receptor (TCR) after forming a peptide-major histocompatibility complex (pMHC). This enabled the identification of neoantigens in the peptide segment from which the mutation originated. Although neoantigens have a higher immunogenicity than tumor-associated antigens, the efficacy of tumor neoantigens is relatively limited. In July 2017, 2 clinical studies of tumor neoantigens simultaneously reported that tumor neoantigens stimulated an anti-tumor immune response in melanoma patients and maintained long-term progression-free survival in stage III melanoma patients; however, in the majority of stage IV melanoma patients, the use of neoantigens led to tumor progression^15,16^. Another 2 clinical studies on tumor neoantigens in glioblastoma suggested that tumor neoantigen vaccines had limited efficacy in some glioblastoma patients^22,23^. The immune response stimulated by neoantigens needs to be improved, and current research on neoantigens remains in the preclinical stage. A study aimed to increase the immunogenicity of neoantigens by exploring new drug delivery systems for neoantigens^24^. However, the ability of T cells to recognize tumor antigens is the most important factor determining an effective immune response. There is no systematic study reporting residue substitution for the modification of neoantigens to improve their immunogenicity. Here, wild-type neoepitopes obtained from a bioinformatics screening were modified by residue substitution, and their affinities were confirmed by bioinformatics analyses. Then, the affinities and stabilities of these neoepitopes were analyzed, and the immunogenicity of wild-type and altered neoepitopes were compared *in vitro* to determine whether the altered neoepitopes were more suitable for immunotherapy of tumors than wild-type neoepitopes.

## Materials and methods

### Clinical information of a patient with advanced gastric adenocarcinoma

A 72-year-old female patient was diagnosed with gastric adenocarcinoma, HER2 negative, and clinical stage IIIC (pT4aN3M0) in November 2016 and subsequently received radical gastrectomy followed with 1 cycle of adjuvant chemotherapy. In October 2017, the patient was diagnosed with left supraclavicular lymph node metastasis and received several courses of chemotherapy, alone or combined with targeted therapy. To enroll in a neoantigen vaccine clinical trial, the patient provided written informed consent for the collection of peripheral blood and paraffin-embedded tumor tissue from primary surgical resection of the stomach for selecting neoepitopes. This study was approved by the Ethics Committee of Fujian Cancer Hospital (Approval No. 2017-043-02).

### Analysis of the raw data of next-generation sequencing and preliminary screening of tumor neoepitopes

DNA was isolated from the patient’s peripheral blood and paraffin-embedded tumor tissues using a Qiagen DNA blood mini kit and Qiagen DNA FFPE (Qiagen, Hilden, Germany), respectively. Whole exome capture libraries were constructed from 1 µg DNA using the BGI Exome Capture V4 Probe (BGI, Shenzhen, China). The libraries were sequenced with 150 nucleotide pair-end reads using the Hiseq4000 platform (Illumina, San Diego, CA, USA).

The patient’s samples were sent to BGI for next-generation sequencing, and raw data from the next-generation sequencing were stored in a computer system called ubuntu16.04.1 LTS for further analysis. Somatic mutations were identified using whole-exome sequencing data from tumor and matched blood samples (as normal references). Cutadapt was used to filter low quality bases or adapter sequences. High quality reads were mapped to the human reference genome (hg38) using BWA^25^, and sorted by chromosomal coordinates with SAMtools^26^. Bamdst was used to analyze the depth of sequencing. The HLA typing of tumor samples was performed using OptiType^27^. Somatic mutations were identified using Mutect2 of GATK (Genome Analysis Toolkit)^28^. MuPeXI was used to analyze neoepitopes from somatic mutations^17^. The 9-mer peptides were generated for neoantigen predictions.

### Bioinformatics analyses of wild-type epitopes and altered epitopes of neoantigens

The most important characteristic of neoantigens is that they are not consistent with the amino acid sequence of normal proteins in the body. Tumor epitopes with the same sequence as those of normal proteins were therefore first excluded, and the neoepitopes in the preliminary screening were filtered. The filter conditions were set as follows: mutant affinity score > 0.5 and normal affinity score < 0.0001. Other conditions were as follows: allele frequency > 0.5%, mutation was set to SNV, proteome peptide match was set to No, with a priority score > 0. The UniProt database (https://www.uniprot.org/) was used to identify glycosylated sites in neoepitopes, and neoepitopes with glycosylated sites were excluded. The cleavage sites of neoepitopes were analyzed by Netchop (http://tools.iedb.org/netchop/)^29–31^, and neoepitopes with 3 consecutive probability scores of cleavage higher than 0.8 were excluded. Proteasomal cleavage/TAP transport/MHC class I combined predictor (http://tools.iedb.org/processing/)^32,33^ was used to calculate the affinity between each neoepitope and TAP, and neoepitopes with a TAP affinity score ≤ 0 were excluded. T cell class I pMHC immunogenicity predictor (http://tools.iedb.org/main/tcell/)^34^ was used for immunogenicity analyses, and neoepitopes with a score ≤ 0 were excluded. Then, the remaining epitopes were evaluated by P1Y (the first amino acid was replaced by tyrosine) and P2L modification (the second amino acid was replaced by leucine). IEDB (prediction method: consensus) was used to score neoepitopes and altered neoepitopes, and the altered neoepitopes with IEDB rank > 1.0 were excluded. BLAST alignment (https://blast.ncbi.nlm.nih.gov/Blast.cgi) was performed on the qualified altered neoepitopes to determine whether the altered neoepitopes had exactly the same sequence as the normally expressed proteins in the human body, and the altered neoepitopes with the same sequence as the normally expressed proteins were excluded. Netchop was used to analyze the cleavage sites of altered neoepitopes, and altered neoepitopes with 3 consecutive probability scores of cleavage > 0.8 were excluded. Proteasomal cleavage/TAP transport/MHC class I combined predictor was used to calculate the affinities between various altered neoepitopes and TAP, and altered neoepitopes with a TAP affinity score ≤ 0 were excluded. Finally, altered neoepitopes were analyzed using the T Cell class I pMHC immunogenicity predictor, and altered neoepitopes with a score ≤ 0 were excluded. According to the ranking of HLA-A0201 affinities of altered neoepitopes, the top 3 altered neoepitopes were selected and compared with wild-type neoepitopes All peptides were synthesized by Genscript (Piscataway, NJ, USA). The purity of all peptides was > 98%, as confirmed by high-pressure liquid chromatography.

### Detections of affinities and stabilities

The T2 cells were placed in a 24-well plate at a density of 1 × 10^6^ cells/well; 50 µg epitope and 3 µg β2M (Merck, Kenilworth, NJ, USA) were then added and incubated in 1 mL fetal bovine free (FBS)-free RPMI1640 medium (Invitrogen, Carlsbad, CA, USA) at 37 °C for 18 h. T2 cells were washed twice with phosphate-buffered saline (PBS), treated with 20 µL PE-HLA-A2 fluorescent antibody (BD Biosciences, San Jose, CA, USA), incubated in the dark for 30 min, and then washed with PBS once. The MFI (mean fluorescence intensity) of HLA-A0201 molecules on the T2 cell surface was detected by flow cytometry (BD Biosciences). The formula used for calculating the fluorescence index was as follows: FI = (MFI of the epitope group – MFI of the control group)/MFI of the control group. The positive control for affinity detection was the HIV-1 peptide (ILKEPVHGV).

T2 cells were cultured with 50 µg of each epitope peptide and 3 µg β2M for 18 h in 1 mL FBS-free RPMI1640 medium at 37 °C in a 24-well plate using 1 × 10^6^ cells/well. T2 cells were then incubated with 5 µg/mL brefeldin A (Abcam, Cambridge, UK) at 37 °C for another 1 h after washing twice with PBS. The T2 cells were then incubated for 0, 6, 12, 18, and 24 h after washing once with PBS, stained with 20 µL PE-HLA-A2 in the dark for 30 min, and washed with PBS. The MFI of the HLA-A0201 molecules in T2 cells was detected by flow cytometry. The results are expressed as DC_50_, which is the time required for 50% dissociation of the pMHC complex stabilized at t = 0 h.

### Induction of neoepitope-specific cytotoxic T lymphocytes (CTLs)

To elicit neoepitope-specific CD8^+^ T cell responses, peripheral blood mononuclear cells (PBMCs) were isolated from healthy HLA-A0201 volunteer donors using lymphocyte separation medium (MP Biomedicals, Burlingame, CA, USA). This protocol was approved by the Ethics Committee of Fujian Cancer Hospital (Approval No. SQ2017-032-01). PBMCs were seeded into a 6-well plate and incubated for 2 h. Adherent cells were then induced to differentiate into dendritic cells (DCs) with 581 medium (Corning, NY, USA) in the presence of 100 ng/mL recombinant human GM-CSF (Mitenyi Biotec, Bergisch Gladbach, Germany) and 50 ng/mL recombinant human IL-4 (Mitenyi Biotec). DCs were sensitized with 10 µg/mL neoepitope, with a preparation without peptide serving as the control. After 24 h, 10 ng/mL IL-6 (Miltenyi Biotec), 10 ng/mL IL-1β (Miltenyi Biotec), 1 µg/mL CD40L (Miltenyi Biotec), and 10 ng/mL TNF-α (Mitenyi Biotec) were added for DC maturation. Next, naïve T cells were co-cultured for 7 days with DCs at a responder: stimulator ratio of 10:1. Medium was then replaced by half-fresh medium containing IL-2 (200 IU/mL, Jiangsu Kingsley Pharmaceutical, Nanjing, China) every 3 days. After another stimulation with neoepitope-loaded DCs for 7 days, CD8 MicroBeads (Mitenyi Biotec) were used to isolate positive CD8^+^ T cells for subsequent experiments.

Detection of CD8^+^ T cell proliferation stimulated by different neoepitopes

The naïve CD8^+^ T cells were adjusted to 1 × 10^7^ cells/mL and subjected to carboxyfluorescein succinimidyl ester (CFSE; Invitrogen) staining. The CFSE concentration was 1 µg/mL. Naïve CD8^+^ T cells were incubated at 37 °C in the dark for 5 min. CFSE-labeled naïve CD8^+^ T cells were incubated in 5 times the volume of 581 medium containing 5% FBS (PAN-Seratech, Aidenbach, Germany) in the dark for 2.5 min, centrifuged at 1,500 rpm for 5 min, and resuspended in 581 medium. The naïve CD8^+^ T cells were co-cultured with DCs with different neoepitopes at a ratio of 10:1 (naive CD8^+^ T cells: 1 × 10^6^ cells: DCs: 1 × 10^5^ cells). After co-culturing with DCs with different neoepitopes for 2 consecutive times, 7 days for each time, T cells were stained with 20 µL PerCP-CD3 fluorescent antibody (BD Biosciences) and 5 µL APC-CD8 fluorescent antibody (BD Biosciences) in the dark for 30 min. The CFSE abundance on the surface of T cells was detected by flow cytometry to determine the proliferation of T cells.

### ELISA assay

The level of IFN-γ or TNF-α in the culture supernatants was detected using commercially available ELISA kits (Thermo Fisher Scientific, Waltham, MA, USA). Briefly, 96-well plates were coated with 100 µL/well capture antibody in coating buffer overnight. Plates were washed with wash buffer 3 times, 200 µL/well ELISA Diluent (1×) was added, and plates were incubated for 1 h. After washing with wash buffer twice, 100 µL/well of supernatants were added, incubated for 2 h, and washed with wash buffer 3 times, followed by addition of 100 µL/well antibodies against human IFN-γ/TNF-α. Plates were incubated for 1 h and then washed with wash buffer 3 times, followed by addition of 100 µL/well streptavidin-horseradish peroxidase conjugate. The plates were then incubated for 30 min and washed with wash buffer 3 times, followed by addition of 1× TMB. After incubation for 15 min, 100 µL/well stop solution was added to each well, and the optical density was measured using a microplate reader at 450 nm.

### Intracellular perforin staining

T cells and T2 cells loaded with wild-type neoepitopes were co-cultured at a ratio of 10:1 for 24 h, incubated in the presence of 5 µg/mL brefeldin A for 1 h, and washed with PBS once. The cells were stained with 20 µL PerCP-CD3 fluorescent antibody and 5 µL APC-CD8 fluorescent antibody in the dark for 30 min, followed by fixation with 100 µL Reagent A from a Intrasure Kit (BD Biosciences) in the dark for 5 min. Subsequently, the cells were permeabilized and stained with 50 µL Reagent B from the Intrasure Kit and 20 µL FITC-perforin fluorescent antibody (BD Biosciences) in the dark for 30 min. After washing with PBS once, the expression of perforin in T cells was detected by flow cytometry.

### Cytotoxicity assay

The amount of lactate dehydrogenase (LDH) released from target cells incubated with neoepitope-specific T cells was measured using the CytoTox 96^®^ Non-Radioactive Cytotoxicity Assay Kit (Promega, Madison, WI, USA). T2 cells loaded with neoepitopes were used as target cells at 1 × 10^4^ cells/well in 96-well plates. Neoepitope-specific CD8^+^ T cells stimulated by DCs presenting with neoepitopes were co-cultured with T2 cells loaded with neoepitopes at an effector/target ratio of 20:1, 10:1, or 5:1 in a 96-well plate at 37 °C. After 4 h, 50 µL of supernatant was transferred from each well to another 96-well plate, and 50 µL of CytoTox 96^®^ reagent (Promega) was added to each well. The plates were then incubated in the dark for 30 min. After adding stop solution at 50 µL/well, the plates were analyzed using a microplate reader at a wavelength of 490 nm. The killing percentage of antigen-specific T cells to T2 cells loaded with neoepitopes was calculated according to the following formula: killing percentage = (experimental release – target spontaneous release – effector spontaneous release)/(target maximum release – target spontaneous release) × 100%.

### Statistical analysis

The results are expressed as the mean ± standard deviation (SD). Multi-group comparisons were analyzed by one-way analysis of variance using Prism 5 software (GraphPad, San Diego, CA, USA). For comparisons between 2 groups, the *t*-test was used for statistical analysis. *P* < 0.05 was considered statistically significant.

## Results

### Identification of neoepitopes of gastric cancers by DNA sequencing and bioinformatics

**Figure 1:**
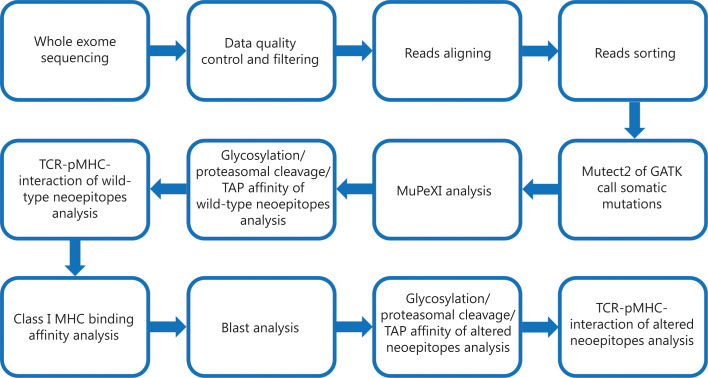
Flow chart of this study.

The flow chart of this study is shown in **Figure 1**. Bamdst was used to detect the average sequencing depth and coverage of tumor and normal samples in the exon regions. The results showed that the average sequencing depth of samples was 861.86× for tumor samples and 331.81× for normal samples (**Supplementary Table S1**). The coverage of tumor sample sequencing data ≥ 100× was 95.16%, and that of normal sample sequencing data ≥ 100× was 86.19% (**Supplementary Table S1**). The HLA typing of the patient showed HLA-A0201, which was confirmed with OptiType analysis of the next-generation sequencing results of the tumor samples (**Supplementary Table S2**).

The somatic mutations of neoepitopes of gastric cancer tissues obtained from next-generation sequencing analyses were analyzed by MuPeXI software. To prevent the negative selection of T cells through cross-recognition, the screening conditions were set as follows: mutant affinity score > 0.5, normal affinity score < 0.0001, allele frequency > 0.5%, mutation = SNV, and proteome peptide match = no, priority score > 0. In total, 16 neoepitopes from the gastric cancer patient were obtained (**Supplementary Table S3**).

The UniProt database showed that there was no glycosylation site in the neoepitopes from the primary screening (**Supplementary Table S4**), and they also did not show 3 consecutive high probability cleavage sites according to Netchop (**Supplementary Table S5**). The 5 neoepitopes, mutCWC22, mutABCA2, mutEXOC2, mutSP140, and mutSTOM, were selected according to their affinities to TAP and TCR by proteasomal cleavage/TAP transport/MHC class I combined predictor analyses and the T cell class I pMHC immunogenicity predictor assay (**Supplementary Tables S6 and S7**). These neoepitopes were analyzed with IEDB after the peptides were altered by P1Y and P2L. The results showed that the affinities between the altered neoepitopes and HLA-A0201 molecules were improved, when compared with that of the original neoepitopes, except for P1Y of mutABCA2 (**Supplementary Table S8**). BLAST results showed that there was no consistent sequence between the altered neoepitopes and the normal proteins in the body (**Supplementary Table S9**), and all altered neoepitopes could be used for further analysis according to Netchop, proteasomal cleavage/TAP transport/MHC class I combined predictor analysis, and T cell class I pMHC immunogenicity predictor analysis (**Supplementary Tables S10–S12**). According to the ranking of HLA-A0201 affinities of altered neoepitopes, the top 3 altered neoepitopes were mutABCA2_L2_ (F**L**GITATVV) mutSP140_Y1_ (**Y**LLPVTCGV) mutSTOM_L2_ (S**L**IISVDGV) (underlined amino acids were changed by genetic mutations. Bold: altered amino acids).

### The altered neoepitopes have higher affinity for HLA-A0201 and higher stability of the pMHC complex than wild-type neoepitopes

**Figure 2:**
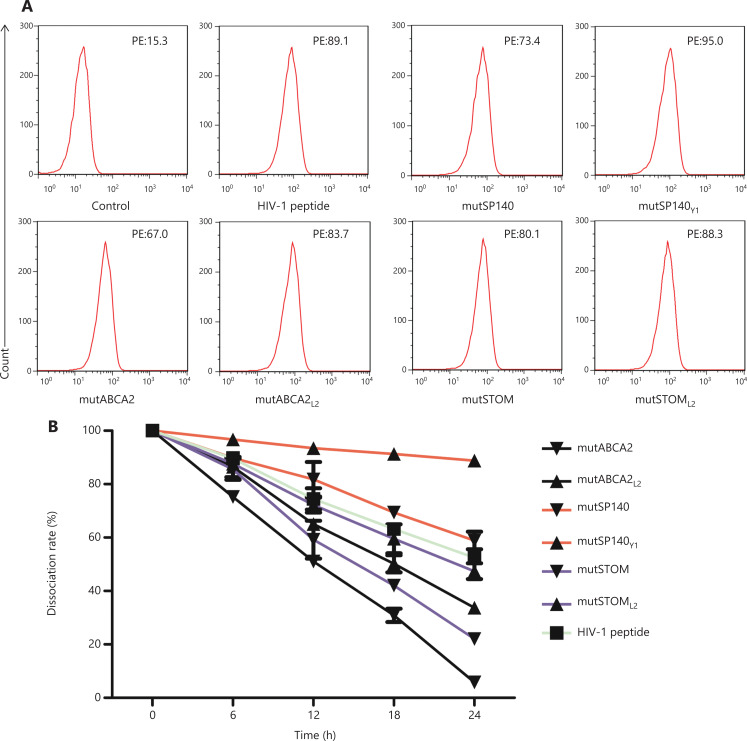
Affinity and stability detection of neoepitopes. (A) Affinity between different neoepitopes and HLA-A0201 molecules. Flow cytometry data are representative of 3 independent experiments. (B) Stability of different neoepitopes and HLA-0201 molecules after forming pMHC complexes. The dissociation rate was calculated as follows: dissociation rate = [mean PE fluorescence with given peptide (t = 0, 6, 12, 18, 24 h) – mean PE fluorescence without peptide (t = 0, 6, 12, 18, 24 h)]/[mean PE fluorescence with a given peptide (t = 0 h) – mean PE fluorescence without peptide (t = 0 h)]. Data represent the mean ± SD of 3 independent experiments.

The T2 affinity assay showed that the affinities of the altered neoepitopes (mutABCA2_L2_ FI: 4.49 ± 0.42, mutSP140_Y1_ FI: 5.20 ± 0.57, mutSTOM_L2_ FI: 4.71 ± 0.41) were higher than those of wild-type neoepitopes (mutABCA2 FI: 3.32 ± 0.36; mutSP140 FI: 3.79 ± 0.50; and mutSTOM FI: 4.27 ± 0.52) (**Figure 2A**). The stabilities of the altered neoepitopes (mutABCA2_L2_, mutSP140_Y1_, and mutSTOM_L2_) were also higher than those of wild-type neoepitopes (mutABCA2, mutSP140, and mutSTOM) (**Figure 2B**). Of the 3 altered neoepitopes, mutSP140_Y1_ had the highest affinity (**Table 1**). The DC_50_ of mutABCA2 was < 18 h, whereas the DC_50_ of mutABCA2_L2_ was > 18 h, and that of mutSP140 and mutSP140_Y1_ was > 24 h (**Table 1**). However the stability results showed that mutSP140_Y1_ was more stable than mutSP140 (**Figure 2B**). The DC_50_ of mutSTOM was < 18 h, whereas the DC_50_ of mutSTOM_L2_ was > 18 h; mutSP140_Y1_ had the highest stability (**Table 1**).

**Table 1:** Affinity prediction and detection results of wild-type neoepitopes and altered neoepitopes

Position of peptide	Sequences	Prediction algorithms-IEDB	FI^a^	DC_50_^b^
mutABCA2	FIGITATVV	3.7	3.32 ± 0.36	<18 h
mutABCA2_L2_	FLGITATVV	1.0	4.49 ± 0.42	>18 h
mutSP140	PLLPVTCGV	2.1	3.79 ± 0.50	>24 h
mutSP140_Y1_	YLLPVTCGV	0.2	5.20 ± 0.57	>24 h
mutSTOM	SVIISVDGV	4.6	4.27 ± 0.52	<18 h
mutSTOM_L2_	SLIISVDGV	1.0	4.71 ± 0.41	>18 h
HIV-1 peptide	ILKEPVHGV	1.8	4.76 ± 0.41	>24 h

^a^FI = (average PE fluorescence with the given peptide – average PE fluorescence without peptide)/(average PE fluorescence without peptide). ^b^DC_50_ is defined as the time required for 50% dissociation of the pMHC complex stabilized at t = 0 h.

### DC cells with altered neoepitopes activate T cells and facilitate the proliferation of T cells

**Figure 3:**
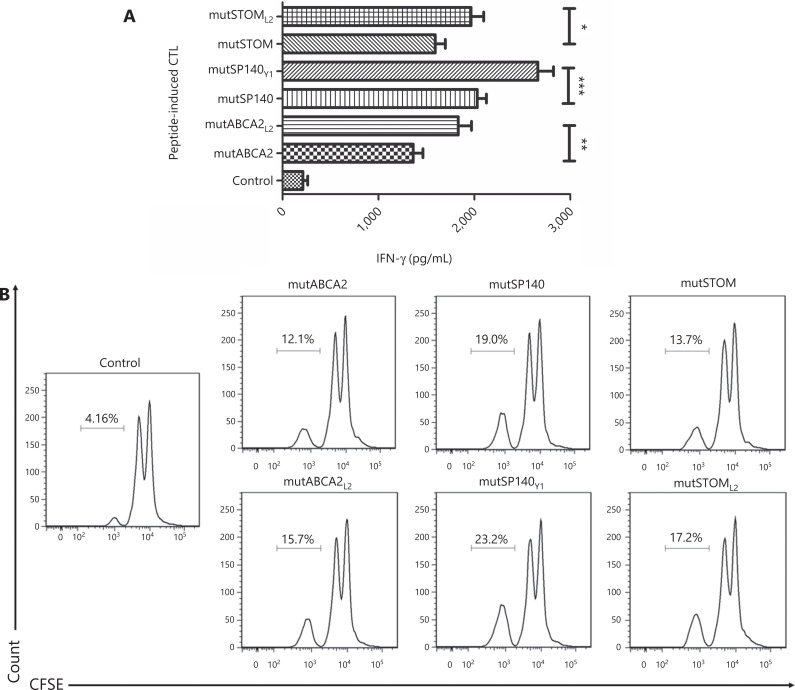
The effect of different neoepitopes on T cells (A) IFN-γ secretion in T cells stimulated by dendritic cells (DCs) with different neoepitopes. Data represent the mean ± SD of 3 independent experiments. *P < 0.05; **P < 0.01; ***P < 0.001. (B) Proliferation of T cells stimulated by DCs with different neoepitopes. Flow cytometry data are representative of 3 independent experiments.

To determine whether altered neoepitopes induced a strong cytotoxic T cell response, the supernatant from altered neoepitope-loaded DC cells co-cultured with T cells was subjected to ELISA for detection of IFN-γ. The results showed that the altered neoepitopes (mutABCA2_L2_, mutSP140_Y1_, and mutSTOM_L2_) stimulated T cells to secrete more IFN-γ than the wild-type neoepitopes (mutABCA2, mutSP140, and mutSTOM) (*P* < 0.05) (**Figure 3A**). The proliferation of specific CD8^+^ T cells was higher in cultures treated with DCs loaded with altered neoepitopes than in those loaded with wild-type neoepitopes (mutABCA2_L2_: 16.47% ± 1.59% *vs.* mutABCA2: 12.53% ± 1.79%, *P* < 0.05; mutSP140_Y1_: 23.43% ± 1.17% *vs.* mutSP140: 18.87% ± 0.81%, *P* < 0.05; mutSTOM_L2_: 17.70% ± 1.14% *vs.* mutSTOM: 14.03% ± 0.76%, *P* < 0.05) (**Figure 3B**).

### The altered neoepitope-specific CTLs effectively inhibited neoepitope-loaded T2 cells by secreting IFN-γ, TNF-α, and perforin

**Figure 4:**
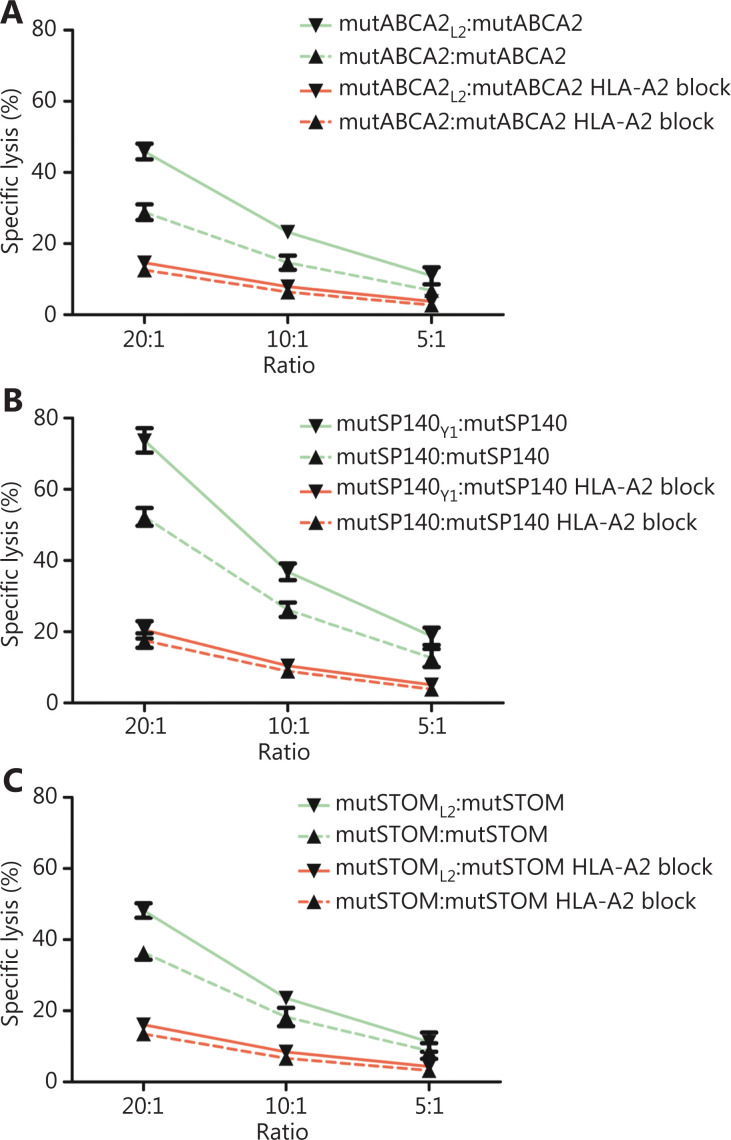
The cytotoxicity of neoepitope-specific cytotoxic T cells (CTLs) stimulated by wild-type and altered neoepitopes on target cells. (A) Comparison of the cytotoxicity of mutABCA2-specific CTLs and mutABCA2_L2_-specific CTLs on T2 cells loaded with mutABCA2. Data represent the mean ± SD of 3 independent experiments. (B) Comparison of the cytotoxicity of mutSP140-specific CTLs and mutSP140_Y1_-specific CTLs on T2 cells loaded with mutSP140. Data represent the mean ± SD of 3 independent experiments. (C) Comparison of the cytotoxicity of mutSTOM-specific CTLs and mutSTOM_L2_-specific CTLs on T2 cells loaded with mutSTOM. Data represent the mean ± SD of 3 independent experiments.

T2 cells loaded with wild-type neoepitopes (mutABCA2, mutSP140, and mutSTOM) were used as target cells to evaluate the cytotoxicity of neoepitope-specific CTLs. The LDH release assay showed that the altered neoepitope (mutABCA2_L2_, mutSP140_Y1_, mutSTOM_L2_)-specific CTLs killed a greater number of T2 cells loaded with wild-type neoepitopes (mutABCA2, mutSP140, mutSTOM) than wild-type neoepitopes (mutABCA2, mutSP140, mutSTOM)-specific CTLs (**Figure 4**).

**Figure 5:**
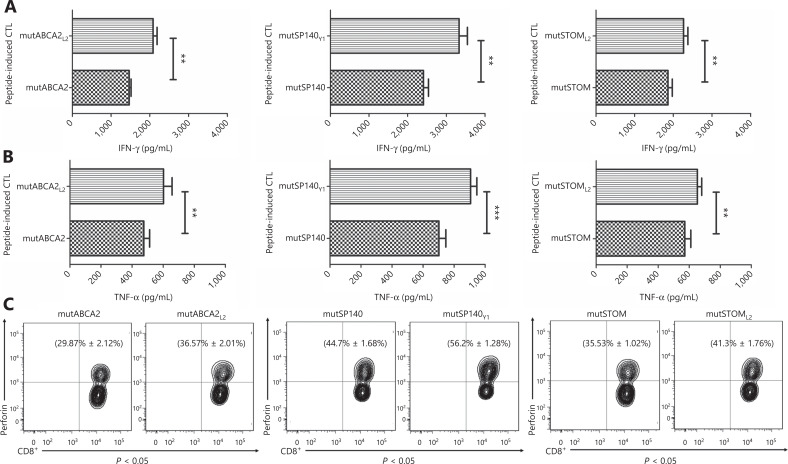
Recognition and activation of neoepitope-specific cytotoxic T cells (CTLs) stimulated by wild-type neoepitopes and altered neoepitopes on T2 cells loaded with neoepitopes. (A) IFN-γ expression in wild-type neoepitope-specific CTLs and altered neoepitope-specific CTLs. Data represent the mean ± SD of 3 independent experiments. *P < 0.05; **P < 0.01; ***P < 0.001. (B) TNF-α expression in wild-type neoepitope-specific CTLs and altered neoepitope-specific CTLs. Data represent the mean ± SD of 3 independent experiments. *P < 0.05; **P < 0.01; ***P < 0.001. (C) Perforin expression in wild-type neoepitope-specific CTLs and altered neoepitope-specific CTLs. The flow cytometry data are representative of 3 independent experiments. Data represent the mean ± SD of 3 independent experiments.

The ELISA assay was used to detect the levels of IFN-γ and TNF-α in the supernatant of T cells co-cultured with target cells, to confirm the cytotoxicity of neoepitope-specific CTLs. The results showed that CTLs stimulated by altered neoepitopes (mutABCA2_L2_, mutSP140_Y1_, and mutSTOM_L2_) released more IFN-γ and TNF-α than CTLs stimulated by wild-type neoepitopes (mutABCA2, mutSP140, and mutSTOM) (*P* < 0.05) (**Figure 5A and 5B**). Intracellular cytokine staining showed that perforin expression was higher in CTLs stimulated by altered neoepitopes than in CTLs stimulated by wild-type neoepitopes (mutABCA2_L2_: 36.57% ± 2.01% *vs.* mutABCA2: 29.87% ± 2.12%, *P* < 0.05; mutSP140_Y1_: 56.20% ± 1.28% *vs.* mutSP140: 44.70% ± 1.68%, *P* < 0.05; mutSTOM_L2_: 41.30% ± 1.76% *vs.* mutSTOM: 35.53% ± 1.02%, *P* < 0.05) (**Figure 5C**).

## Discussion

The occurrence of cancer is related to the accumulation of a large number of genetic mutations^35–37^. Cancer-associated genetic mutations include the functionally acquired mutations of oncogenes and the functionally missing mutations of tumor suppressor genes; however, most of them are randomly generated “passenger” mutations with unclear functions. Therefore, tumors from different patients have different mutation profiles, and the individual biological characteristics of the tumor lead to different responses to the same treatment regimen. In the era of precision therapy, the best treatment strategy for cancer is to select the appropriate drugs for each patient according to the specific molecular characteristics of the tumor.

Advances in sequencing technology and the continuous decrease of sequencing costs have led to the wide use of genotype analysis using sequencing data of tumor tissues and normal cells in clinical practice. It is used not only to determine the appropriate targeted drugs for each patient according to DNA/RNA sequencing of the tumor tissue, but also to predict the prognoses of patients using immune checkpoint inhibitors such as PD-1 antibody according to the frequency of gene mutations in the tumor tissue. The development of gene sequencing technology has therefore facilitated the application of individualized immunotherapy.

The clinical application of PD-1 antibodies is increasingly used in anti-cancer immunotherapy in recent years because of its definite target and efficacy for activating T cells. However, the anticancer therapeutic response in clinical practice is limited. This can be attributed to a low tumor mutation burden^38,39^ or microsatellite stability^40^.

The mutated genes in tumor tissues produce abnormal proteins. These aberrant proteins or peptides can be recognized by the immune system to induce anti-tumor immune responses. Neoantigens are abnormal proteins encoded by genes in tumor cells with point mutations, deletion mutations, or gene fusion, which are different from the proteins expressed in normal cells. These neoantigens are digested to form peptide fragments (neoepitopes) that are processed by DCs and finally presented to T cells to induce cancer-specific CTLs.

The discovery of specific immune responses induced by neoantigens led to the clinical application of immunotherapies targeting neoantigens. Mutations in KRAS, an oncogene, are frequent in different kinds of cancers and contribute to tumorigenesis and disease progression. CD8^+^ T-cells against mutant KRAS G12D obtained from tumor-infiltrating lymphocytes (TILs) of a patient with metastatic colorectal cancer were reinfused at 1.11 × 10^11^ into the patient, resulting in regression of metastatic colon cancer after therapy^41^. This supported the feasibility of immunotherapy targeting neoantigens. In 2017, 2 Phase I clinical trials showed that neoantigens can be used as peptide vaccines or mRNA vaccines to induce tumor regression in melanoma^15,16^. This made possible the clinical application of neoantigen vaccines for solid tumors. Ott et al.^15^ showed that of 6 melanoma patients who received a neoantigen peptide vaccine, 4 did not have a recurrence within 20–32 months. The other 2 patients showed tumor regression after adjuvant therapy (anti-PD-1 antibody). Vaccination induced strong multi-functional CD4^+^and CD8^+^ T-cell responses in patients with high risk melanomas. Sahin et al.^16^ used an RNA-based poly-neoepitope vaccine to mobilize the immunity of patients with advanced melanoma; the results showed that among the 13 vaccinated patients, 8 had no recurrence within 12–23 months. One patient had a complete response to vaccination in combination with PD-1 blockade therapy. These results suggested that neoantigens produced synergistic effects in combination with PD-1 antibody.

In addition to melanoma, solid tumors such as breast cancer showed clinical regression after adoptive transfer of TILs specifically targeting neoantigens. A female with estrogen receptor-positive and ERBB2 receptor tyrosine kinase-negative metastatic breast cancer who was refractory to multiple lines of chemotherapy received transfer of TILs targeting the mutant proteins SLC3A2, KIAA0368, CADPS2, and CTSB combined with checkpoint blockade, which led to complete durable regression for over 22 months^42^. This demonstrated that neoantigens derived from clonal mutations could be important targets of adoptive cell therapy.

Neoantigens are identified mainly by bioinformatics analysis of tumor SNVs; therefore, it is necessary to increase sequencing depth to improve the detection of SNVs^43^. In this study, the average sequencing depth of tumor samples was > 800×, which was considerably higher than the conventional whole-exome sequencing depth of 100–200×^43,44^, and the sequencing raw data were converted into BAM files for analysis. A large number of HLA-A0201 high affinity neoantigens were detected in the gastric cancer tissue of this patient. However, the epitope from the mutated gene only differed by 1 amino acid from that of the original unmutated gene. Therefore, it is likely that the neoantigen-specific T cells also recognized the epitope from the unmutated gene^45^. In this study, after preliminary screening, the neoepitopes with an affinity score of the original epitope < 0.0001 were reserved to avoid negative selection. Here, we selected 3 suitable tumor neoepitopes with high affinity for HLA, namely, mutABCA2 (FIGITATVV), mutSP140 (PLLPVTCGV), and mutSTOM (SVIISVDGV). We showed that these neoepitopes activated and promoted the proliferation of T cells with cytotoxic effects against target cells loaded with neoepitopes. The altered neoepitopes with a P1Y or P2L amino acid substitution induced a stronger immune response than the wild-type neoepitopes. This indicated that residue substitution could be used to enhance the immunogenicity of neoepitopes by increasing the affinity of the neoepitopes for HLA molecules, as well as the stability of the pMHC complexes formed by the neoepitopes and HLA molecules, which are important for immunogenicity^46,47^. Affinity is an important determinant of immunogenicity^48,49^. A higher affinity between the antigen epitope and the HLA molecule resulted in a higher abundance of pMHC complexes, which provides a strong first stimulus signal for T cells. Therefore, increasing affinity improved the immunogenicity of the epitope. Studies showed that the stable binding time between T cells and antigen-presenting cells (APCs) was positively associated with the activation of T cells^50^. If the bond is broken, the activation of T cells is affected to some extent, indicating that the maintenance of long-term contact between T cells and APCs, namely the long-term immune synapse, is an important factor for T cell activation. The poor stability of the pMHC complex may indirectly affect the contact between APCs and T cells, which can impair the activation of T cells. The results of an early study on alterations of the melanoma tumor-associated antigen, gp100_209–217_ (ITDQVPFSV), also induced a better immune response^51^. The present results indicated that neoantigens could be modified by replacing residues to improve their immunogenicity, thereby providing an experimental foundation for the modification of neoantigens in future clinical practice.

Our study confirmed that altered neoepitopes induced a stronger antitumor immune response than wild-type neoepitopes. However, this needs to be confirmed using *in vivo* experiments. For example, altered neoepitopes and wild-type neoepitopes can be inoculated into humanized mice to compare the immunogenicity of the 2 types of neoepitopes *in vivo*. The study of antigens needs to be transformed from *in vitro* to *in vivo* to provide valuable information for future clinical applications.

Although neoantigens can induce stronger immunogenicity than tumor-associated antigens, they also have a few disadvantages. Some tumor cells do not carry neoantigens, which can lead to immune escape. These issues could be resolved using the following methods: (1) identification of clonal neoantigens in the tumor. Compared with the subclonal neoantigens that can only cover part of the tumor clones, clonal neoantigens that can cover most of the tumor cells have a higher clinical utility^52,53^. Immunotherapy with clonal neoantigens can eliminate tumor cells to the maximum extent. (2) Neoantigens encoded by tumor-driven mutated genes are used as targets for attack. Tumor progression is mostly driven by tumor-driven mutated genes. For example, the unmutated TP53 gene can induce cell death in response to DNA damage^54^. TP53 loses function after mutation; therefore, the cells carrying TP53 mutation can be used as targets for attack, which can effectively kill tumor cells with higher malignancy. For tumor patients with driver mutations, neoantigen therapy with a driver mutation source can inhibit tumor growth more effectively. (3) Multiple neoantigens can be used simultaneously to ensure a greater coverage of tumor subclones and to decrease the escape of tumor subclones^55^.

## Conclusions

Amino acid residue substitution improved the affinity between neoepitopes and HLA molecules and increased the stability of the pMHC complexes formed by the neoepitopes and HLA molecules. Altered neoepitopes had a higher immunogenicity than wild-type neoepitopes. The present results may lead to a new strategy of personalized immunotherapy through the modification of neoantigens in clinical practice.
