# Supplementary material for: Residue substitution enhances the immunogenicity of neoepitopes from gastric cancers
Source: Cancer Biol Med. 2021 Nov 24;18(4):1053–65. doi: 10.20892/j.issn.2095-3941.2021.0022 (PMC8610152; doi:10.20892/j.issn.2095-3941.2021.0022)
Supplement: Supplementary file 1 [file cbm-18-1053-s001.pdf]

Supplementary materials

Table S1 Sequencing depth and coverage of tumor and normal samples

	Tumor sample	Normal sample
Average depth	861.86×	331.81×
Coverage (> 0×)	99.86%	99.65%
Coverage (≥ 4×)	99.71%	99.42%
Coverage (≥ 10×)	99.42%	98.97%
Coverage (≥ 30×)	98.68%	97.15%
Coverage (≥ 100×)	95.16%	86.19%

Table S2 HLA typing test results of gastric cancer sample sequencing in patients with gastric cancers

Software	Tumor sample
OptiType	HLA-A*02:01:01:01

Table S3 Preliminary screening results of neoepitopes in gastric cancers

Gene symbol	Gene ID	Norm peptide	Normal affinity score	Amino acid change	Mutant peptide	Mutant affinity score	Allele frequency	Priority score
FRYL	ENSG00000075539	IIFDLLSVG	2.03E-23	G/A	IIFDLLSVA	0.999054641	0.07	7
TPP2	ENSG00000134900	DLKEEFTEA	1.09E-08	D/N	NLKEEFTEA	0.990092438	0.036	4
CBLC	ENSG00000142273	ELGQVPLSA	3.40E-05	Q/E	ELGEVPLSA	0.720310035	0.033	2
DPP8	ENSG00000074603	PLLDLFQAT	6.39E-08	P/S	SLLDLFQAT	0.999645519	0.015	1
CWC22	ENSG00000163510	RLKDETLQP	7.23E-15	K/M	RLMDETLQP	0.954587851	0.013	1
BANP	ENSG00000172530	ITQDSEGNL	5.24E-28	T/M	IMQDSEGNL	0.81252482	0.014	1
SOGA1	ENSG00000149639	ELEDQNKLL	6.18E-15	E/K	KLEDQNKLL	0.992739359	0.011	1
ABCA2	ENSG00000107331	FIGITATVA	4.85E-08	A/V	FIGITATVV	0.68793868	0.015	1
AL121768	ENSG00000165521	ELDSEIEQE	9.09E-61	E/V	ELDSEIEQV	0.998283928	9.24E-03	1
VN1R4	ENSG00000228567	KIAQTLRAM	3.97E-07	R/H	KIAQTLHAM	0.746683177	0.011	1
ACTN1	ENSG00000072110	NLNTAFDVA	3.85E-05	D/H	NLNTAFHVA	0.510998226	0.016	1
EXOC2	ENSG00000112685	RIEALRELL	7.32E-08	R/I	RIEALIELL	0.707132961	9.16E-03	1
SP140	ENSG00000079263	PLLPVTCGG	4.76E-80	G/V	PLLPVTCGV	0.954976407	6.69E-03	1
CD38	ENSG00000004468	RKDCSNNPV	6.83E-70	K/M	RMDCSNNPV	0.874242242	6.46E-03	1
NUDT12	ENSG00000112874	YFLHPPMPA	0.000482523	H/P	YFLPPPMPA	0.712795488	7.28E-03	1
STOM	ENSG00000148175	SVTISVDGV	7.32E-05	T/I	SVIISVDGV	0.98767836	5.16E-03	1

Table S4 Screening results of glycosylation sites of neoepitopes

Gene symbol	Mutant peptide	Glycosylation
FRYL	IIFDLLSVA	No
TPP2	NLKEEFTEA	No
CBLC	ELGEVPLSA	No
DPP8	SLLDLFQAT	No
CWC22	RLMDETLQP	No
BANP	IMQDSEGNL	No
SOGA1	KLEDQNKLL	No
ABCA2	FIGITATVV	No
AL121768	ELDSEIEQV	No
VN1R4	KIAQTLHAM	No
ACTN1	NLNTAFHVA	No
EXOC2	RIEALIELL	No
SP140	PLLPVTCGV	No
CD38	RMDCSNNPV	No
NUDT12	YFLPPPMPA	No
STOM	SVIISVDGV	No

Table S5 Digestion analysis results of neoepitopes

Gene symbol	Mutant peptide	3 sequential sites with high possibility of proteasomal cleavage
FRYL	IIFDLLSVA	No
TPP2	NLKEEFTEA	No
CBLC	ELGEVPLSA	No
DPP8	SLLDLFQAT	No
CWC22	RLMDETLQP	No
BANP	IMQDSEGNL	No
SOGA1	KLEDQNKLL	No
ABCA2	FIGITATVV	No
AL121768	ELDSEIEQV	No
VN1R4	KIAQTLHAM	No
ACTN1	NLNTAFHVA	No
EXOC2	RIEALIELL	No
SP140	PLLPVTCGV	No
CD38	RMDCSNNPV	No
NUDT12	YFLPPPMPA	No
STOM	SVIISVDGV	No

Table S6 Analysis results of affinity between neoepitopes and the antigen peptide transporter

Gene symbol	Mutant peptide	Affinity to TAP
FRYL	IIFDLLSVA	−0.17
TPP2	NLKEEFTEA	−0.18
CBLC	ELGEVPLSA	−0.39
DPP8	SLLDLFQAT	−0.29
CWC22	RLMDETLQP	0.12
BANP	IMQDSEGNL	0.42
SOGA1	KLEDQNKLL	0.39
ABCA2	FIGITATVV	0.03
AL121768	ELDSEIEQV	−0.03
VN1R4	KIAQTLHAM	0.20
ACTN1	NLNTAFHVA	−0.22
EXOC2	RIEALIELL	0.42
SP140	PLLPVTCGV	0.07
CD38	RMDCSNNPV	0.13
NUDT12	YFLPPPMPA	−0.23
STOM	SVIISVDGV	0.20

Table S7 Affinity analysis results of neoepitopes and the T cell receptor

Gene symbol	Mutant peptide	TCR affinity
CWC22	RLMDETLQP	0.02232
BANP	IMQDSEGNL	−0.05731
SOGA1	KLEDQNKLL	−0.25255
ABCA2	FIGITATVV	0.27643
VN1R4	KIAQTLHAM	−0.02634
EXOC2	RIEALIELL	0.26437
SP140	PLLPVTCGV	0.03628
CD38	RMDCSNNPV	−0.22618
STOM	SVIISVDGV	0.0934

Table S8 Affinity analysis results of altered neoepitopes and HLA-A0201 molecules

Gene symbol	Mutant peptide	Neoantigen	1Y	2L
		IEDB	IEDB	IEDB
CWC22	RLMDETLQP	6.0	1.4	–
ABCA2	FIGITATVV	3.7	3.9	1.0
EXOC2	RIEALIELL	10.0	3.9	3.6
SP140	PLLPVTCGV	2.1	0.2	–
STOM	SVIISVDGV	4.6	1.6	1.0

Table S9 BLAST analysis results of homology between the altered neoepitopes and human normal proteins

Gene symbol	Altered mutant peptide	Similarity (100%) to protein exist in human
CWC22	RLMDETLQP(P1Y)	No
ABCA2	FIGITATVV(P2L)	No
EXOC2	RIEALIELL(P2L)	No
SP140	PLLPVTCGV(P1Y)	No
STOM	SVIISVDGV(P2L)	No

Table S10 Digestion analysis results of altered neoepitopes

Gene symbol	Altered mutant peptide	3 sequential sites with high possibility of proteasomal cleavage
CWC22	RLMDETLQP(P1Y)	No
ABCA2	FIGITATVV(P2L)	No
EXOC2	RIEALIELL(P2L)	No
SP140	PLLPVTCGV(P1Y)	No
STOM	SVIISVDGV(P2L)	No

Table S11 Analysis results of affinity between altered neoepitopes and the antigen peptide transporter

Gene symbol	Altered mutant peptide	TAP affinity
CWC22	RLMDETLQP(P1Y)	0.04
ABCA2	FIGITATVV(P2L)	0.01
EXOC2	RIEALIELL(P2L)	0.40
SP140	PLLPVTCGV(P1Y)	0.11
STOM	SVIISVDGV(P2L)	0.18

Table S12 Affinity analysis results of altered neoepitopes and the T cell receptor

Gene symbol	Altered mutant peptide	TCR affinity
CWC22	RLMDETLQP(P1Y)	0.02232
ABCA2	FIGITATVV(P2L)	0.27643
EXOC2	RIEALIELL(P2L)	0.26437
SP140	PLLPVTCGV(P1Y)	0.03628
STOM	SVIISVDGV(P2L)	0.0934
